# A Case of Castleman’s Disease with a Marked Infiltration of IgG4-Positive Cells in the Renal Interstitium

**DOI:** 10.3390/diagnostics14050476

**Published:** 2024-02-23

**Authors:** Erika Sawada, Yuya Shioda, Kohki Ogawa, Takatsugu Iwashita, Yuko Ono, Hajime Hasegawa, Akito Maeshima

**Affiliations:** Department of Nephrology and Hypertension, Saitama Medical Center, Saitama Medical University, Kawagoe 350-8550, Japan

**Keywords:** idiopathic multicentric Castleman disease (iMCD), IgG4-related disease (IgG4-RD), amyloidosis, membranous nephropathy

## Abstract

Multicentric Castleman’s disease (MCD) is a benign lymphoproliferative disorder with heterogenous clinical symptoms, and involves systemic organs in addition to lymph nodes. Herein, we present the case of a 55-year-old man with MCD characterized by an extensive infiltration of IgG4+ plasma cells in the kidneys. The patient presented to our hospital with a high fever and diarrhea. On admission, laboratory analysis revealed anemia, renal dysfunction (eGFR 30 mL/min/1.73 m^2^), polyclonal gammopathy (IgG 7130 mg/dL), elevated serum IgG4 level (2130 mg/dL), and increased C-reactive protein (8.0 mg/dL). An enlargement of lymph nodes in the axillary, mediastinal, para-aortic, and inguinal regions was observed on abdominal computed tomography. Axillary lymph node biopsy revealed interfollicular expansion due to dense plasma cell infiltration. Renal biopsy demonstrated significant plasma cell infiltration into the tubulointerstitium. Immunohistochemical analysis showed a 40% IgG4-positive/IgG-positive plasma cell ratio, meeting the diagnostic criteria for an IgG4-related disease. Amyloid A deposition was observed along vessel walls, and immunofluorescence analysis indicated granular positivity of IgG and C3 along the glomerular capillary wall. Elevated levels of interleukin-6 (21 pg/mL) and vascular endothelial growth factor (VEGF; 1210 pg/mL) were noted. Based on these findings, and the histological finding of the lymph node biopsy, idiopathic MCD was diagnosed. Corticosteroid monotherapy was only partially effective. Subsequently, tocilizumab administration was initiated, leading to sustained remission, even after discontinuation of prednisolone. Due to the diverse responses to steroid therapy and the varying prognoses observed in MCD and IgG4-related disease, it is essential to carefully diagnose MCD by thoroughly assessing the organ distribution of the disease, its response to steroid therapy, and any additional pathological findings.

## 1. Introduction

Castleman’s disease is a lymphoproliferative disorder with distinct histopathological features that was first described by Benjamin Castleman in 1956 [1]. The main subtypes are unicentric Castleman disease (UCD), characterized by a single enlarged lymph node, and multicentric Castleman disease (MCD), which is further classified into idiopathic MCD (iMCD), human herpes virus-8-related (HHV8-MCD), and POEMS-MCD. iMCD contains the subcategories iMCD-TAFRO and iMCD-NOS [2,3]. The pathogenesis is characterized by the overproduction of IL-6, although the mechanisms by which IL-6 levels become elevated remain unclear [4]. Clinical manifestations of MCD vary, with systemic symptoms including fever, night sweats, and fluid retention due to hyper-IL-6emia. Severe manifestations producing “life-threatening cytokine storms” and multi-organ failure have been reported [5,6]. Anemia and polyclonal γ-globulinemia are common features of MCD.

iMCD is primarily diagnosed based on pathological histological findings, encompassing distinct subtypes such as the “vascular hyperplasia type” marked by regressive changes in germinal centers and significant neovascularization, the “plasma cell type” characterized by germinal center hyperplasia and a notable increase in plasma cells, and a mixed type displaying overlapping features of both [2]. One essential diagnostic criterion involves the recognition of enlargement in two or more lymph nodes. Positron emission tomography–computed tomography (PET-CT) plays a crucial role not only in identifying enlarged lymph nodes, but also in distinguishing between iMCD and lymphoma [2]. To reach a diagnosis of CD, it is necessary to exclude diseases that mimic iMCD, including autoimmune diseases such as systemic lupus erythematosus (SLE) and rheumatoid arthritis, as well as infectious diseases and malignancies.

Patients with UCD often remain asymptomatic, and complete recovery typically follows the surgical removal of the affected lymph nodes. Conversely, managing MCD predominantly involves IL-6 inhibitors, regardless of disease severity [7]. The Food and Drug Administration has approved the use of an anti-IL-6 antibody, siltuximab, for iMCD in the United States [8]. In Japan, tocilizumab, an anti-interleukin-6 (IL-6) receptor antibody, is an approved therapeutic option for iMCD. In cases resistant to IL-6 inhibition, alternative therapies, including corticosteroids, rituximab, cyclosporine, thalidomide, and sirolimus, have been investigated [9,10]. Although corticosteroid treatment yields transient relief in approximately 50% of cases, recurrence is common. Prolonged corticosteroid use entails risks of adverse events, such as opportunistic infections, impaired glucose tolerance, and bone fragility, underscoring the importance of careful treatment selection [11].

IgG4-related disease (IgG4-RD) is a fibroinflammatory disorder characterized by elevated serum IgG4 levels and organ swelling caused by IgG4-positive plasma cells. The main pathological features of IgG4-RD include lymphoid cell infiltration, fibrosis, obliterative phlebitis, and eosinophil infiltration [11,12]. IgG4-RD can affect nearly all organs, including the pancreas, biliary system, salivary glands, periorbital tissues, kidneys, lungs, lymph nodes, aorta, and skin [13,14]. Presenting with diverse clinical manifestations, IgG4-RD often mimics malignant diseases, infectious disorders, and inflammatory conditions. Patients with IgG4-RD often have concurrent allergic diseases such as atopy, bronchial asthma, and chronic rhinosinusitis [15]. Treatment varies based on the affected organ and the extent of fibrosis. The primary therapeutic agent is glucocorticoids, which are beneficial in the majority of cases [16].

Elevated IL-6 levels stimulate polyclonal B-cell activation, leading to increased serum IgG4 levels. In cases of MCD, diagnostic criteria for IgG4-RD are frequently met, manifesting as lymph node enlargement with Castleman-like histopathological findings [17]. Despite their similarities, MCD and IgG4-RD differ in terms of pathophysiology, with varying responses to treatment. While moderate glucocorticoid doses effectively control IgG4-RD, many MCD cases require IL-6 inhibitors for disease activity control [5,9,18]. Comprehensive assessment of clinical symptoms, serological findings, and pathological histology is essential for accurate differentiation between these conditions. Herein, we report a unique case of iMCD that was difficult to distinguish from IgG4-RD because of a strikingly elevated IgG4 serum concentration and abundant IgG4-positive cells in the kidney.

## 2. Case Presentation

A 55-year-old man was referred to our hospital for a high fever and diarrhea. On admission, his laboratory data revealed anemia (hemoglobin 8.7 g/dL), renal dysfunction (serum creatinine 1.96 mg/dL), and proteinuria (urinary protein 0.44 g/gCr) (Table 1). Other abnormal laboratory results were hypoalbuminemia (albumin 2.5 g/dL), hyperproteinemia (total protein 10.9 g/dL), abnormal serum levels of immunoglobulins (IgG 7132 mg/dL, IgG4 2130 mg/dL, IgA 238 mg/dL, IgM 93 mg/dL), elevated soluble IL-2 receptors (3991 U/mL), and increased inflammatory markers (CRP 4.98 mg/dL, ESR 80 mm/hour). Pertinent negative results were noted in white cell count (7500/μL), platelet count (271,000/μL), hepatic profiles (AST 11 IU/L, ALT 8 IU/L), electrolytes (sodium 135 mmol/L, potassium 4.7 mmol/L), and complement (C3 99 mg/dL, C4 16 mg/dL). Immunofixation electrophoresis was negative for M protein. The free light chain showed no abnormality with a κ/λ ratio of 1.57, indicating that multiple myeloma was unlikely. The workups for infectious diseases showed negative results for HHV-8 and tuberculosis. Further workups for autoimmune diseases were low positive for antinuclear antibodies (speckled 1:40). Notably, IL-6 was as high as 21 pg/mL (reference range, ≤7 pg/mL). VEGF was also high at 1044.4 pg/mL (reference range, 143.1–658.1 pg/mL).

**Figure 1:**
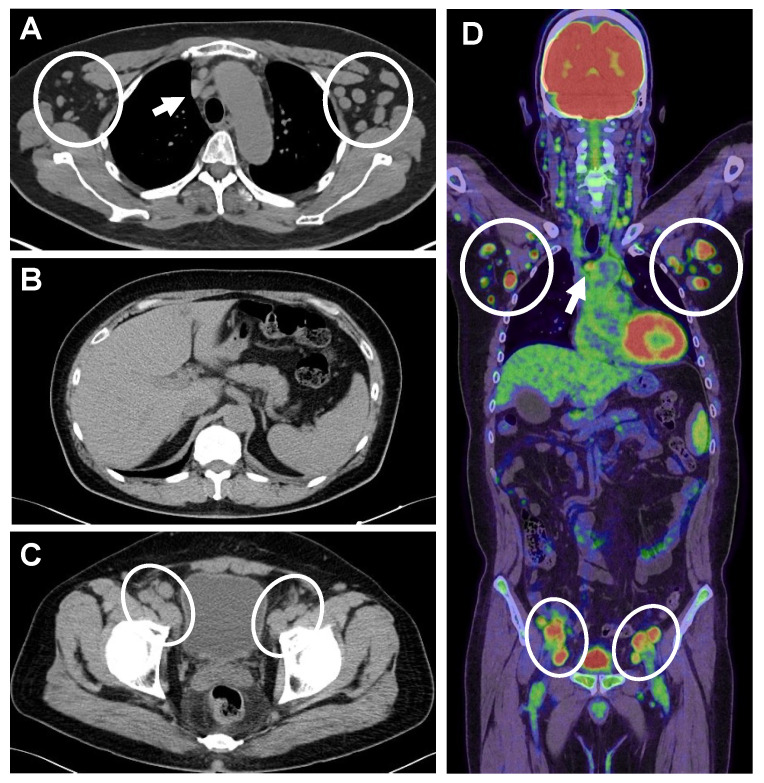
PET-CT imaging. (**A**) Enlargement of axillary (circles) and mediastinal lymph nodes (arrow). (**B**) No hepatosplenomegaly or ascites are seen. (**C**) Inguinal lymph node enlargement (circle). (**D**) FDG accumulation consistent with enlarged lymph nodes (circles).

Chest X-ray revealed a cardiothoracic ratio of 51% with no abnormal shadows in lung fields. The presence of multiple swollen lymph nodes in various locations, including axillary, hilar, mediastinum, para-aortic, and inguinal regions, was observed on abdominal CT. PET-CT imaging confirmed the enlargement of axillary and mediastinal lymph nodes (Figure 1A), absence of hepatosplenomegaly or ascites effusion (Figure 1B), enlargement of the iliac artery area and inguinal lymph nodes (Figure 1C), and FDG accumulation in large lymph nodes (Figure 1D).

**Figure 2:**
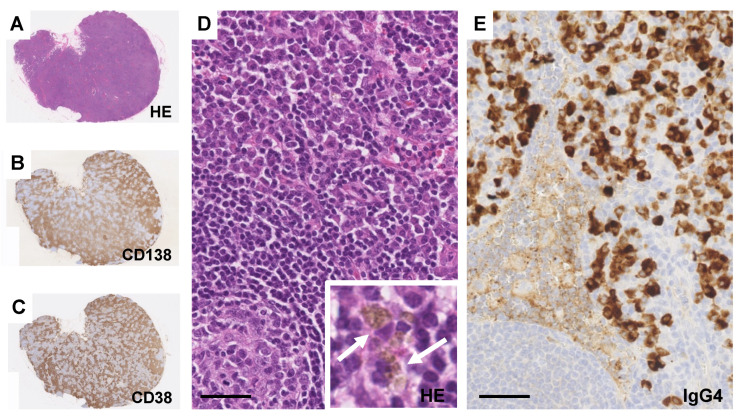
Light microscopic findings of the lymph node. (**A**–**C**) An increase in plasma cells is visible between the enlarged follicles (**A**), with equal proportions of CD38-positive (**B**) and CD138-positive (**C**) plasma cells. (**D**) A sheet-like plasmacytosis in the interfollicular area. Bar = 50 μm. Insert picture shows scattered hemosiderin-laden macrophages (arrows). (**E**) Immunostaining of IgG4. Bar = 50 μm.

**Figure 3:**
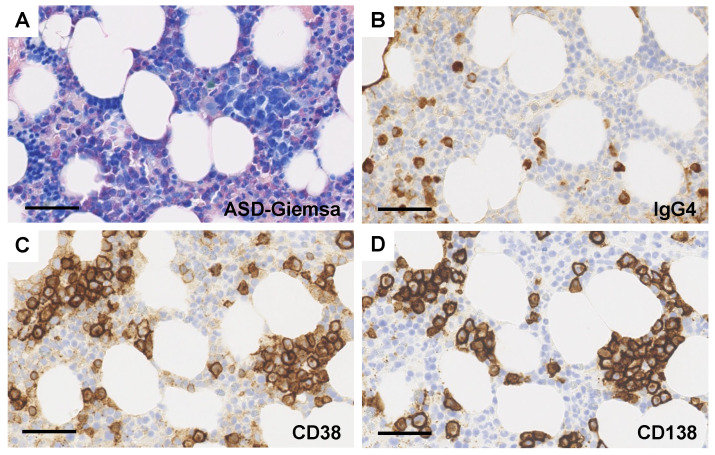
Aspiration biopsy of the bone marrow. (**A**) ASD-Giemsa staining. Bar = 50 μm. (**B**–**D**) Immunostaining of IgG4 (**B**), CD38 (**C**), and CD138 (**D**). Bars = 50 μm.

**Figure 4:**
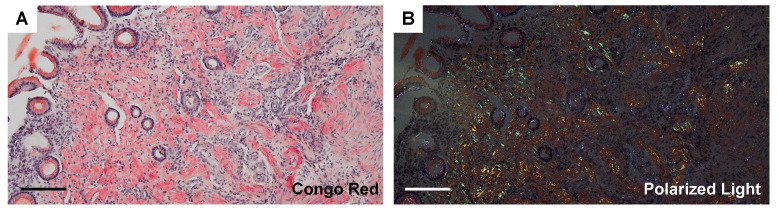
Gastric mucosa biopsy. (**A**) Congo-red staining. Bar = 100 μm. (**B**) Polarized microscopy. Congo-red-positive deposition with apple-green polarization is visible in the lamina propria. Bar = 100 μm.

**Figure 5:**
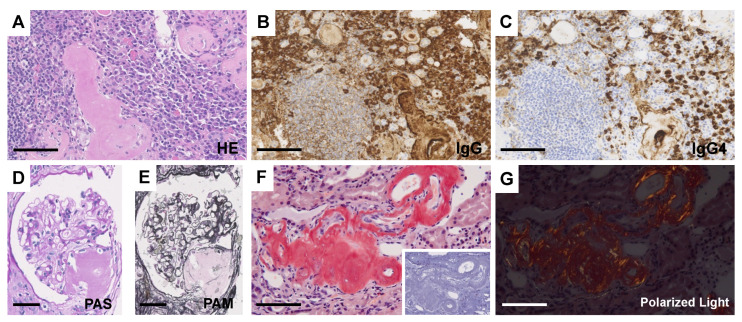
Light microscopic findings of the kidney. (**A**) HE staining. Dense plasmacytic infiltration is focally seen in the interstitium. (**B**,**C**) Immunostaining of IgG (**B**) and IgG4 (**C**). Bars = 100 μm. (**D**) PAS staining. (**E**) PAM staining. Nodular deposition of amorphous material is shown in the vascular pole of some glomeruli. Bars = 50 μm. (**F**) DFS staining. Amorphous deposition in small arteries to arterioles is positive for DFS staining and disappeared after permanganate treatment (insert). Bar = 100 μm. (**G**) Apple-green birefringence is recognized by polarized microscopy. Bar = 100 μm.

**Figure 6:**
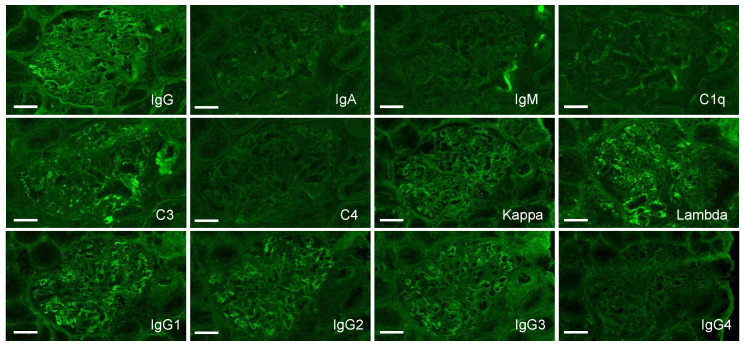
Immunofluorescence study of the kidney. Relatively faint, granular positivity of IgG and C3 is shown on a glomerular capillary wall. IgA, IgM, C1q, and C4 are negative. IgG subclass staining shows positivity of IgG1, IgG2, and IgG3; however, IgG4 is negative. Light chain restriction is not observed. Bars = 50 μm.

**Figure 7:**
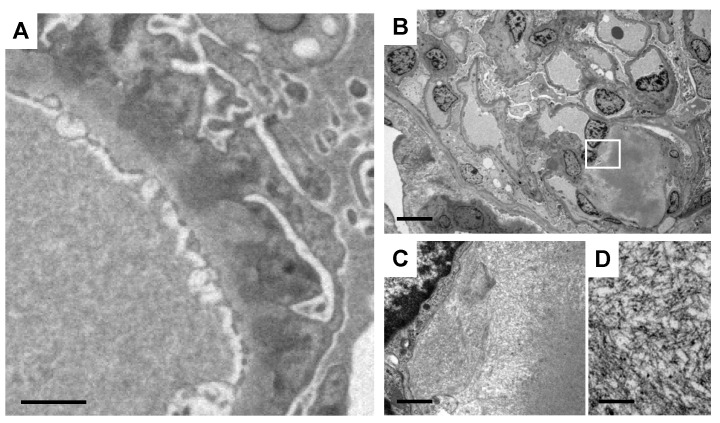
Electron microscopic finding of the kidney. (**A**) Electron-dense deposits of the subepithelial area. Bar = 2 μm. (**B**) An irregular increase in electron density in the mesangial area. Bar = 10 μm. (**C**,**D**) Unbranched, microfibrillar structures with a random arrangement. Bars = 1 μm in (**C**) and 0.5 μm in (**D**).

Biopsies of lymph nodes, kidneys, gastric mucosa, and bone marrow were conducted. The axillary lymph node biopsy revealed an increase in plasma cells between the enlarged follicles, with equal proportions of CD38-positive and CD138-positive plasma cells (Figure 2A–C). Enlarged interfollicular spaces, atrophied germinal centers, mildly increased vasculatures, and highly increased plasmacytes (sheet-like plasmacytosis) were observed (Figure 2D). Hemosiderin-laden macrophages were occasionally seen (insert in Figure 2D). The number of IgG4-positive plasmacytes was 122/HPF on average (Figure 2E). The IgG4/IgG ratio was approximately 50–60%. Aspiration biopsy of the bone marrow revealed a 10% positivity for IgG4 staining in the bone marrow (Figure 3A,B), along with an elevated presence of CD38-positive (Figure 3C) and CD138-positive plasma cells (Figure 3D). Gastric mucosa biopsy showed Congo-red positive deposition (Figure 4A) with apple-green polarization recognized in the lamina propria via polarized microscopy (Figure 4B). The renal biopsy demonstrated a dense plasmacytic infiltration, localized within the interstitium (Figure 5A). Immunohistochemical analysis disclosed a 40% IgG4-positive/IgG-positive plasma cell ratio (Figure 5B,C). Storiform fibrosis and owl-eye-pattern fibrosis were notably absent. Nodular deposition of amorphous material was observed in the vascular pole of certain glomeruli (Figure 5D,E). Amorphous deposition in small arteries to arterioles was positive for DFS staining (Figure 5F) and subsequently disappeared following permanganate treatment (insert in Figure 5F). Apple-green birefringence was recognized using polarized microscopy (Figure 5G). Immunofluorescence analysis revealed a somewhat subdued, granular positivity of IgG and C3 along the glomerular capillary wall. IgA, IgM, C1q, and C4 exhibited negative results. No evidence of light chain restriction was observed. Subsequent examination of IgG subclasses indicated positivity for IgG1, IgG2, and IgG3, while IgG4 was not detected (Figure 6). Electron microscopy revealed the presence of electron-dense deposits in the subepithelial regions (Figure 7A). The mesangial area also exhibited an irregular elevation in electron density (Figure 7B). Amyloid deposition was identified based on the presence of unbranched fibrils, measuring 10 to 12 nm in width and arranged in a random pattern (Figure 7C,D).

**Figure 8:**
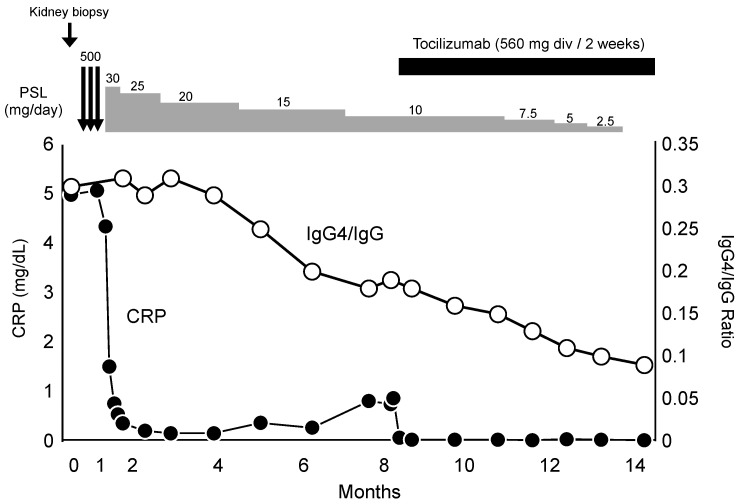
Clinical course of this case. IgG4/IgG ratio (white circles), C-reactive protein (CRP) (black circles). PSL, prednisolone.

According to the patient’s laboratory findings, clinical symptoms, and biopsy results, idiopathic MCD was diagnosed. Following the start of steroid pulse therapy, treatment was continued with prednisolone 30 mg/day as maintenance therapy. A therapeutic response was promptly observed, with a decreasing trend in CRP and IgG4 values. However, upon a reduction in the prednisolone dosage to 10 mg/day, CRP became elevated again. Considering a resurgence of disease activity, tocilizumab was administered every two weeks (8 mg/kg/day) from the 260th day. Subsequently, CRP became negative, and remission was maintained even after discontinuation of prednisolone (Figure 8).

## 3. Discussion

This case fulfilled two primary criteria outlined in the diagnostic guidelines for iMCD: “generalized lymphadenopathy” and “lymph node histopathological findings compatible with the plasmacytic type CD”. Furthermore, laboratory results revealed elevated ESR and CRP, polyclonal hypergammaglobulinemia, and renal dysfunction. Clinical observations included fever and weight loss. HHV-8 was negative, and other infections, autoimmune diseases, and malignant conditions were ruled out. Consequently, a definitive diagnosis of iMCD was made. Corticosteroid monotherapy demonstrated only partial efficacy. A subsequent administration of tocilizumab was initiated, resulting in sustained remission, even after the discontinuation of prednisolone.

Although this case met the diagnostic criteria for iMCD, the patient also exhibited three findings characteristic of IgG4-RD. The first was the elevated level of serum IgG4/IgG ratio. Serum IgG4 elevation in iMCD has been reported to be observed in 73.3% of cases; consequently, the serum IgG4 level is not considered useful for distinguishing between IgG4-RD and iMCD [19,20]. When differentiating between IgG4-RD and iMCD, the IgG4/IgG ratio in both serum and tissue (normal range, <0.05) is considered valuable [20]. However, there are cases of MCD with IgG4/IgG ≥ 0.4, making it challenging to differentiate between IgG4-RD and MCD based solely on the immunostaining of lymph node lesions [21]. In the present case, the IgG4/IgG ratio in the lymph node was approximately 50–60%. Furthermore, the serum IgG4/IgG ratio was elevated at 0.3, which is atypical for MCD.

The second characteristic of IgG4-RD exhibited by the present case was secondary membranous nephropathy. In patients diagnosed with MCD, the co-occurrence of renal diseases is not uncommon, with approximately 25% of MCD patients exhibiting nephritis or renal failure [22,23,24]. Renal diseases associated with CD include small vessel lesions (SVL) associated with thrombotic microangiopathy (TMA) and membranoproliferative glomerulonephritis (MPGN), as well as AA amyloidosis. Although complication of membranous nephropathy is commonly observed in IgG4-RD [25], it is rare in iMCD [23,24,26,27].

The final characteristic of IgG4-RD exhibited by the present case was the lack of an elevated serum IgA level. Elevated IL-6 levels, central to iMCD pathophysiology, are typically linked with increased levels of CRP, ESR, and immunoglobulins. In IgG4-RD, the concentration of IL-6 generally does not increase, suggesting the potential for IL-6 or its related factors to serve as biomarkers for distinguishing between the conditions [21,28]. Among the biomarkers examined to date, IgA has shown promise as being potentially valuable for discriminating between IgG4-RD and iMCD, with an area under the curve (AUC) of 0.96, a cutoff value of 330 mg/dL, sensitivity of 93.2%, and specificity of 93.9% [29].

In MCD cases, elevated IL-6 levels may coexist with increased IgG4, frequently fulfilling diagnostic criteria for IgG4-RD [21,30,31]. In cases lacking clear MCD symptoms like fever, there is a risk of misdiagnosis as IgG4-RD. Even with elevated serum IgG4 or organ infiltration by IgG4-positive plasma cells, persistent CRP positivity or a limited response to steroids suggests considering the possibility of MCD [30]. A previous report demonstrated the case with overlapping features of IgG4-RD and MCD present in a single patient, suggesting that both diseases might be caused by a common pathogenesis [32]. An accurate differentiation requires comprehensive assessment of age, affected organs, IL-6 concentration, serum IgG4/IgG ratio, and pathological observations [19,20]. In 2020, the IgG4-Related Diseases Pathology Research Group in Japan introduced exclusion criteria to differentiate and avoid the misdiagnosis of diseases resembling IgG4-RD [33]. Based on clinical and histopathological findings in patients with iMCD and IgG4-RD, a validation study assessed the IgG4-Related Diseases Exclusion Criteria, demonstrating a sensitivity of 100% and specificity of 93% [34]. In the present case, the presence of persistent positive CRP, sheet-like plasma cell proliferation, and hemosiderin deposition led to the exclusion of IgG4-RD, and a final diagnosis of iMCD was then confirmed.

## 4. Conclusions

In summary, we present the case with iMCD characterized by extensive infiltration of IgG4+ plasma cells in the kidneys. Although iMCD often exhibits characteristics that meet the diagnostic criteria for IgG4-RD, the response to steroid therapy and prognosis differ between iMCD and IgG4-RD. Therefore, the diagnosis of iMCD should be carefully considered, taking into account various factors such as the distribution of organ involvement, response to steroid therapy, and other pathological findings. The present case underscores the heterogeneous nature of MCD pathophysiology, raising questions about potential differences in treatment response, renal prognosis, and overall life expectancy between MCD cases with elevated serum IgG4 levels and those with normal levels. The identification of accurate markers to distinguish between these groups and monitor treatment response would certainly enhance the diagnosis and treatment of this disease subtype.

## Figures and Tables

**Table 1:** Laboratory findings on admission. WBC: white blood cell, RBC: red blood cell, Hb: hemoglobin, Plt: platelet, ESR: erythrocyte sedimentation rate, NAG: *N*-acetyl-β-D-glucosaminidase, β2MG: β2-microglobulin, BJP: Bence Jones protein, TP: total protein, Alb: albumin, AST: aspartate aminotransferase, ALT: alanine aminotransferase, BUN: blood urea nitrogen, Cr: creatinine, eGFR: estimate glomerular filtration rate, UA: uric acid, CRP: C-reactive protein, ANA: antinuclear antibody, sIL-2R: soluble interleukin-2 receptor, HHV-8: human herpesvirus type 8.

Blood Count			Biochemistry			Immunology		
WBC	7500	/μL	TP	10.9	g/dL	IgG	7132	mg/dL
RBC	324 × 10^4^	/μL	Alb	2.5	g/dL	IgG4	2130	mg/dL
Hb	8.7	g/dL	AST	11	U/L	IgA	238	mg/dL
Plt	27.1 × 10^4^	/μL	ALT	8	U/L	IgM	93	mg/dL
ESR	80	mm/h	BUN	28	mg/dL	C3	99	mg/dL
			Cr	1.96	mg/dL	C4	16	mg/dL
Urinalysis			eGFR	29.4	mL/min/1.73 m^2^	ANA	40	times
Specific gravity	1.011		UA	9.8	mg/dL	M protein	(-)	
pH	5.5		Na	135	mEq/L	sIL-2R	3991	U/mL
Sugar	(-)		Cl	106	mEq/L	Amyloid A	35	mg/L
Proteinuria	(±)		K	4.7	mEq/L	IL-6	21.1	pg/mL
Occult blood	(±)		CRP	4.98	mg/dL	VEGF	1044.4	pg/mL
Sediment RBC	1–4	/HPF	Fe	26	μg/dL	HHV-8	(-)	
Urinary Protein	0.44	g/gCr	TIBC	188	μg/dL			
NAG	11.9	IU/L	ferritin	92	ng/mL			
β2MG	3406	μg/L						
BJP	(-)							

## Data Availability

The original contributions presented in the study are included in the article, further inquiries can be directed to the corresponding author.
